# Research on one-dimensional phase change heat transfer characteristics based on instrument compartment structure

**DOI:** 10.1038/s41598-024-67484-x

**Published:** 2024-07-27

**Authors:** Jiabin Zhao, Longbin Liu, Mingkun Li

**Affiliations:** 1 College of Aerospace Science and Engineering, National University of Defense Technology, Changsha, 410073 China; 2 Hunan Provincial Key Laboratory of Aerospace Cross-Domain Flight Vehicle System and Control Technology, Changsha, 410073 China

**Keywords:** One-dimensional phase change heat transfer, Instrument compartment structure, Phase change material, Energy science and technology, Engineering, Physics

## Abstract

To address the issue of electronic equipment failure inside the instrument compartment due to aerodynamic heating during high-speed flight. Combining the heat transfer characteristics of phase change materials, a new instrument compartment structure was proposed as the research subject based on phase change materials. While studying the heat transfer characteristics of this structure, one-dimensional phase change heat transfer theoretical model was constructed based on the Lightfoot integral equation method, and the corresponding analytical solution was obtained. To explore the temperature change law of the instrument compartment structure and verify the rationality of the theoretical model, the new thermal experiment was carried out for the instrument compartment structure. Compared with the aluminum alloy instrument compartment structure, the experimental results show that the instrument compartment structure design based on phase change materials could effectively reduce the temperature of the structure itself, and the experimental data are in good agreement with the theoretical calculation results, which verified the rationality of the theoretical model and provided a scientific basis for the practical application of phase change materials in instrument compartment structures.

## Introduction

Phase Change Materials (PCMs) refer to a class of materials capable of changing their state under heating or cooling conditions, thereby storing or releasing energy. Phase change materials possess advantages such as high heat storage/release capacity, low density, high reliability, and excellent reusability^1,2^. Moreover, during the phase change process, they can absorb or release a significant amount of heat while maintaining constant or nearly constant temperature, leading to their wide application across various fields. In the aerospace domain, numerous scholars have conducted extensive research on thermal management utilizing phase change materials. Among the notable contributions, Vrable and Vrable^3^ proposed a phase change temperature control system for phased array antennas on low Earth orbit satellites, which absorbs a considerable amount of heat during the phase change process to maintain stable temperatures during periods of sunlight and eclipse. Izenson et al.^4^ demonstrated through experiments that phase change material heat storage units could enhance the thermal stability of loop heat pipes in small satellite thermal control systems. Smay et al.^5^ introduced a structural design for phase change material panels for cubic satellite thermal control using aluminum powder direct metal laser sintering. Sanusi et al.^6^ made phase change materials into phase change energy storage components for electronic instrument temperature control devices to achieve desired thermal control effects. Cao et al.^7^ designed a novel integrated thermal protection structure containing phase change materials, demonstrating through simulation and experimentation its superior thermal insulation and heat protection capabilities.

While the application of phase change materials has yielded fruitful results, considerable progress has also been made in the field of phase change heat transfer theory. Hasan et al.^8^ conducted numerical simulations of cyclic melting and freezing of flat-type phase change materials based on one-dimensional phase change heat transfer, with numerical results closely aligned with experimental observations. Costa et al.^9^ used enthalpy formulation and fully implicit finite difference method to analyze the thermal performance of storage systems with and without fins, deriving an empirical formula for the effective thermal conductivity of one-dimensional phase change problems considering the effect of natural convection. Mosaffa et al.^10^ proposed an analytical approach to phase change heat transfer with internal finned plates, establishing a two-dimensional numerical model based on enthalpy formulation to predict the temperature distribution of fins and solid–liquid interfaces during the storage process, with results from both analytical and numerical models showing excellent consistency. Mendonca and Elisan^11^ proposed a new method for addressing unstable enthalpy terms in the heat diffusion equation, demonstrating better agreement with experimental data compared to classical formulas. Tanathep et al.^12^ considered factors such as airflow, conduction, convection, and radiation heat exchange, employing a zonal method to simulate a simplified insulated box heat transfer model, yielding results in good agreement with measurements. Feng et al.^13^ utilized the lattice Boltzmann method with three-dimensional enthalpy formulation to simulate solid–liquid phase change process within porous skeletons, elucidating the heat transfer mechanism of convection phase change with pore scale. Zhou^14^ conducted research on the enhancement of phase change heat transfer and thermal storage performance in porous media through the preparation of composite phase change materials. As research on phase change heat transfer continues to deepen, it significantly enriches the methods for solving phase change heat transfer problems.

Despite numerous simulation and solution methods for phase change heat transfer having been proposed, there are still key scientific issues that remain to be resolved, to which Ye has made significant contributions. In the simulation of the pure solid gallium phase change process, there were issues such as inappropriate definition and calculation of interface error, and unsatisfactory numerical verification and validation. Ye and Arıcı^15^ conducted a two-dimensional verification of the pure gallium melting pore model, re-examined and rigorously demonstrated the definition and calculation method of the interface position error between numerical and experimental results. In light of the existing research not thoroughly exploring the computational methods for the mushy zone constant *A*_*m*_, Ye and Arıcı^16^ explored the relationship between *Am* and *ΔT* through the dimensionless analysis of the melting process of CaCl_2_·6H_2_O, and successfully established a rapid calculation method for *A*_*m*_. Regarding the sources of error, Ye and Arıcı^17^ combined with experimental data from the literature, comprehensively discussed the three-dimensional verification and two-dimensional feasibility, and thoroughly discussed and summarized the discrepancies between the current three-dimensional and two-dimensional modeling of pure solid gallium phase change and the experimental measurement results in the literature. In addition, based on the finite volume enthalpy-porosity method, Ye and Arıcı^18^ explored and resolved three key scientific issues in the modeling of pure solid gallium phase change, discussed innovative findings and fitting correlations of the phase change equilibrium state, and comprehensively demonstrated the dimensionless correlation between the average liquid layer thickness and the global liquid volume fraction.

## Design of instrument compartment structure based on phase change materials

**Figure 1:**
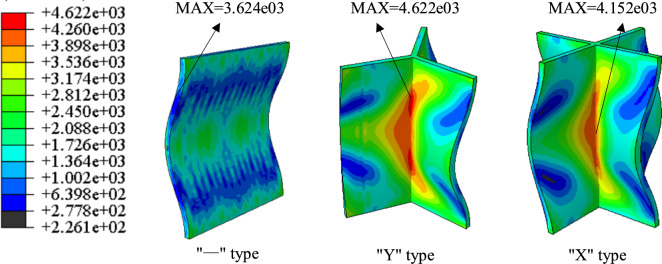
Stress distribution nephogram of simulation results of three structures.

In general, the structural design of an aircraft's instrument compartment should meet the following design requirements: it should not only provide space for accommodating most instrument equipment such as control systems and flight measurement systems, but also possess a streamlined aerodynamic shape capable of withstanding various loads and environmental conditions^19,20^. Considering that the aircraft instrument compartment mainly bears axial loads during flight, the structural design requirements demand a certain load-carrying capacity and sufficient internal space to accommodate electronic equipment. Common structural design forms include the “—” type, “Y” type, and “X” type, etc. Other structural forms are not considered due to potential issues such as increased mass and reduced space. Finite element modeling is carried out for the three structures, where, in addition to the different structural designs, the corresponding load conditions, material parameters, and corresponding thicknesses are set to be the same, as detailed in Table 1. Through finite element simulation, the stress distribution maps and critical load conditions of the three structures can be obtained, as shown in Fig. 1 and Table 2.

**Table 1:** Setting of material parameters and boundary conditions.

Density (kg/m^3^)	Elastic modulus (GPa)	Poisson's ratio	Displacement load (mm)	Thickness (mm)
2810	71.7	0.33	20	8

**Table 2:** Critical load cases of simulation results of three structures.

Type	Maximum stress (MPa)	Critical bearing capacity (KN)
“一” type	3624	254.4
"Y" type	4622	3316
"X" type	4152	4145

**Figure 2:**
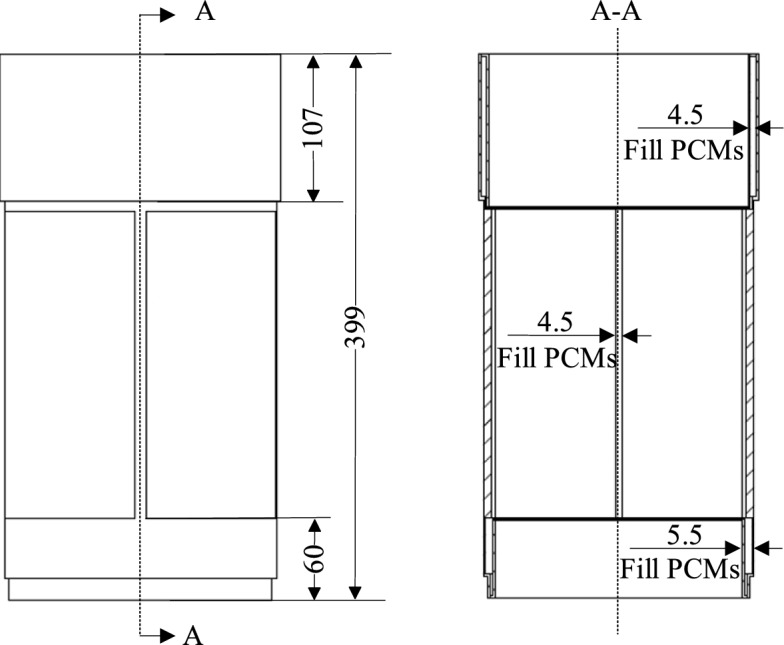
Schematic diagram illustrating the location of phase change material filling.

**Figure 3:**
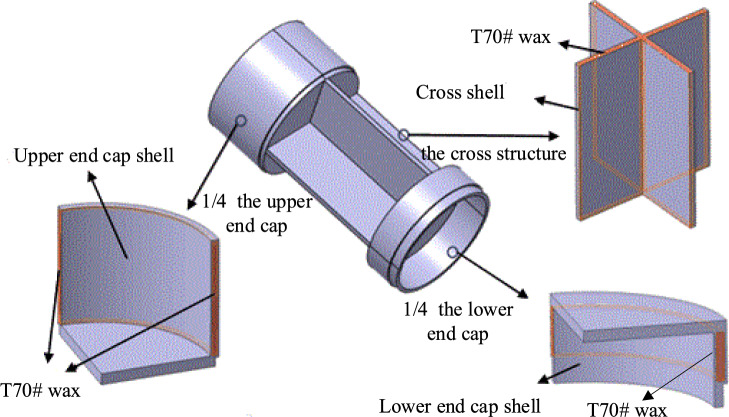
The composition of the instrument compartment structure.

It can be observed from the figures that under the same conditions, the “X” type design possesses the greatest load-bearing capacity, significantly exceeding that of the “–“ type design; the maximum stress of the “X” type design is lower than that of the “Y” type design. Considering that the structure will also need to accommodate slots for filling phase change materials, which will lead to a reduction in load-bearing capacity, the “X” type design with greater load-bearing capacity is selected, also known as the cross structure. Drawing from typical applications of phase change materials in aerospace, and considering the requirements for instrument compartment structural design, this paper proposes a design for instrument compartment structure based on phase change materials. As shown in the figures, Fig. 2 depicts a schematic diagram of the location where phase change materials are filled within the instrument compartment structure, while Fig. 3 illustrates the composition of the instrument compartment structure.

The structural composition of the instrument compartment comprises two main components: phase change materials and a phase change shell. The phase change shell consists of three parts: the upper end cap, the cross structure, and the lower end cap. The upper and lower end caps serve primarily for interconnection with other components, while their internal space accommodates sizable equipment. The central section is designed with a cross layout, tasked with providing requisite space while withstanding axial loads. The upper and lower end caps and the cross structure are treated with grooves internally, filled with phase change materials, and finally sealed and formed by welding. The purpose of this design has two aspects: one is that the phase change material has a lower density, which can be used to reduce weight and does not occupy extra space; the other is to utilize the heat absorption characteristic of the phase change material during the phase change process, to provide a certain degree of thermal protection: when the external temperature rises and the heat is transferred to the inside of the structure, the temperature increases to the phase change temperature, the internal phase change material absorbs a large amount of heat during the phase change, effectively slowing down the rapid rise in temperature, and providing a certain degree of thermal protection to protect the internal equipment.

The 7075 aluminum alloy is distinguished by its lightweight, cost-efficiency, moderate specific strength, and excellent corrosion resistance and toughness, rendering it extensively utilized in the automotive and aerospace industries^21,22^. Given considerations such as load-bearing requirements, corrosion resistance, and production costs, the material selected for the phase change shell of the instrument bay structure is the 7075 aluminum alloy.

Considering that the phase change temperature of the selected phase change material is about 70 ℃, it belongs to low-temperature phase change material. Low temperature phase change materials mainly include inorganic and organic phase change materials, among which inorganic phase change materials have disadvantages such as supercooling, phase separation and corrosiveness. Paraffin in organic phase change materials has the characteristics of stability, commonness and low cost. Some paraffin phase change materials are comprehensively screened according to the requirements of phase change temperature, the phase change material selected in this paper is the 70# special wax from the 70# microcrystalline wax, referred to as T70# wax, with the general chemical molecular formula: C_20_H_50_O_2_. It has a phase change latent heat of 230 kJ/kg and a phase change temperature of approximately 70 ℃. T70# wax boasts advantages such as high phase change latent heat, minimal supercooling phenomenon, low vapor pressure during melting, and resistance to chemical reactions^23,24^. The relevant material properties of 7075 aluminum alloy and T70# wax are presented in Table 3.

**Table 3:** The material parameters for 7050 aluminum alloy and T70# wax.

Material type	7050 Aluminum alloy	T70# wax
Elastic modulus (GPa)	71.7	1.5
Poisson's ratio	0.33	0.37
Density (kg/m^3^)	2810	840
Specific heat capacity (J·kg^−1^ ℃^−1^)	840	2000
Thermal conductivity coefficient (W·m^−1^ ℃^−1^)	130	0.558
Coefficient of thermal expansion (℃^−1^)	2.32 × 10^–5^	8.62 × 10^−5^
Yield limit (MPa)	554	–
Phase transition temperature *T*_*m*_ (℃)	–	70

The instrument bay structure design proposed in this article, based on T70# wax, significantly reduces the structure’s mass. Due to the density difference between 7075 aluminum alloy and T70# wax, compared to a solid instrument bay structure entirely composed of 7075 aluminum alloy, this approach reduces the structure's mass from 5.39 to 1.6942 kg, achieving a mass reduction of 31.43%.

## Numerical analysis

### Constructing theoretical models

**Figure 4:**
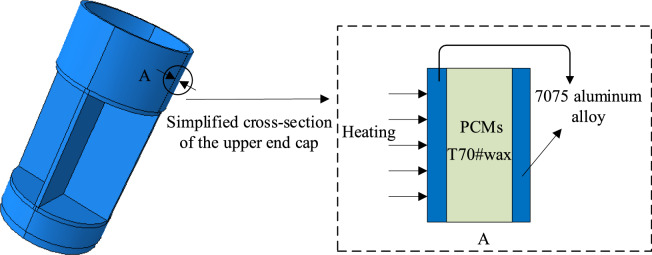
The simplified cross-section of the upper end cap.

The internal structure of the instrument compartment is simplified to a one-dimensional sandwich plate configuration by filling it with T70# wax and employing a thinner filling layer design. As illustrated in Fig. 4, this configuration's distribution across the cross-section is further simplified into a one-dimensional phase change heat transfer model. Unlike the irregularity of the solid–liquid interface in other large-sized filled phase change materials during phase change, this model assumes that the solid–liquid interface is parallel to the shell surface during the phase change process and moves in the same direction as the heat conduction. This implies that over time, the solid–liquid interface changes direction along a determined axis, indicating the one-dimensional movement of the solid–liquid interface.

The heat transfer problem involving phase change entails various complexities, including the movement of the phase change interface, the mushy region between solid and liquid phases, natural convection within the liquid phase, and the volumetric changes of the target material. These complexities imbue the problem with pronounced nonlinear characteristics, rendering the solving process intricate^25,26^. Investigating phase change process necessitates considering numerous intricate factors such as temperature, pressure, thermodynamic properties, and the type of phase change material. Directly solving such problems entails dealing with a plethora of mathematical equations, leading to complex workloads with no guarantee of analytical solutions. Consequently, it is often necessary to simplify theoretical models in the analysis of phase change heat transfer. Simplified models not only elucidate the physical transformation process, facilitating comprehension and analysis, but also make it easier to obtain approximate or numerical solutions. Throughout the entire phase change process, it may be assumed that the phase change duration is extremely short but involves a significant absorption of heat. To streamline numerical analysis, the following assumptions are proposed:

1. The top and bottom of the instrument compartment are designated as adiabatic boundaries.
2. Due to the negligible difference in density between the solid and liquid phases of T70# wax, volume changes are essentially disregarded during the phase change process.
3. The phase change process is rapid, assuming no liquid flow phenomena occur during this change.
4. Throughout the heat transfer process, internal radiation within the instrument compartment is negligibly small, and the influence of gravity is disregarded.

The Lightfoot integral equation method, also known as the moving heat source method, postulates the presence of a moving planar heat source or sink at the solid–liquid interface during the phase change process. Under this assumption, the phase change heat transfer equations can be decomposed into two simpler heat conduction equations: one without a mobile heat source but with unchanged boundary conditions and initial conditions, and the other incorporating a fixed planar heat source with both boundary and initial temperatures set to zero. Based on this premise, this solving approach transforms the phase change heat transfer problem into a transient heat conduction problem. Within the transient heat conduction equation set, various parameters for the solid and liquid phases can be configured, significantly reducing the complexity of the numerical model and mitigating the computational burden of the solution process^27,28^.

**Figure 5:**
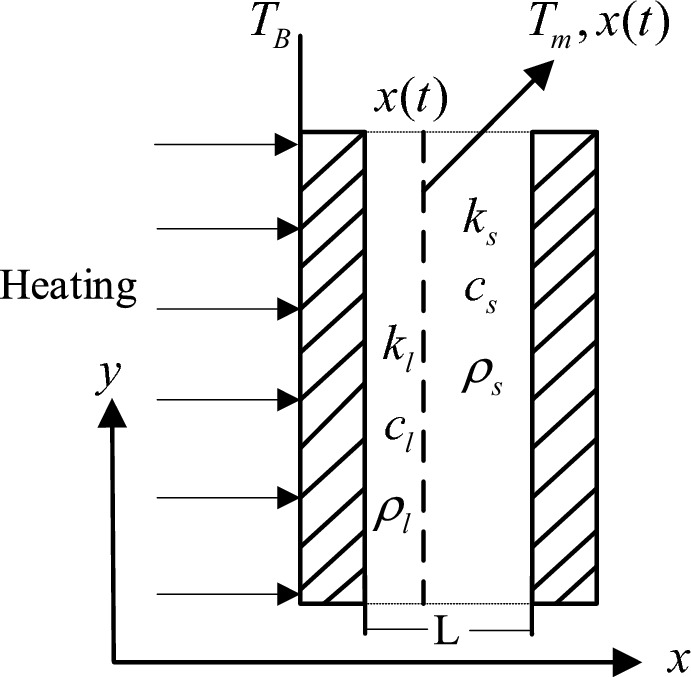
The schematic diagram of one-dimensional phase change heat transfer physical model.

Considering the structural characteristics of the model in this paper, we establish a one-dimensional phase change heat transfer theoretical model based on phase change materials, as illustrated in Fig. 5. In the figure, the shaded regions before and after represent the shell of the filler material, while the middle section represents the phase change material being filled, with a thickness denoted by *L*. The arrow on the left indicates heating from the left side, and the dashed line in the middle represents the solid–liquid phase boundary.

Distinguishing between melting and solidification: In the melting process of phase change materials, there is considered to be a mobile thermal sink within the interior, whereas in the solidification process, there is deemed to be a mobile heat source within the material. Considering the heat transfer characteristics of the proposed instrument compartment structural model in this paper, when simplified into a one-dimensional phase change heat transfer model, it can be assumed that during the endothermic melting process of the phase change material, there exists a mobile thermal sink within the material, at the position *x*(*t*), there is a moving planar thermal sink moving parallel to the shell surface along the direction of heat transfer. Simplifying into a one-dimensional heat conduction equation yields the following heat transfer control equation for the phase change material^29^:

1 $$k\frac{{\partial^{2} T(x,t)}}{{\partial x^{2} }} + \rho \Delta h\frac{dx(t)}{{dt}}\delta [x - x(t)] = \rho c\frac{\partial T(x,t)}{{\partial t}}$$

In the equation: *ρ* Is the density; *k* is the thermal conductivity; *c* is the specific heat capacity; the subscript "*s*" represents the solid phase; the subscript "*l*" represents the liquid phase; *δ*[*x*-*x*(*t*)] is the Dirac function, and its corresponding expression is as follows:

2 $$\delta [x,x(t)] = \left\{ \begin{gathered} 0 , \quad x \ne x(t) \hfill \\ 1 , \quad x = x(t) \hfill \\ \end{gathered} \right.$$

The initial conditions and boundary conditions for this model are as follows:

3 $$T(x,t) = T_{B} \quad x = 0 ,t > 0$$

4 $$T(x,t) = T_{i} \quad 0 < x < L ,t = 0$$

5 $$\frac{\partial T(x,t)}{{\partial x}} = 0 , \quad x = L,t > 0$$

In the equation: *T*_*B*_ denotes the boundary temperature for heat transfer, which is influenced by the temperature field imposed by the surroundings. *T*_*i*_ represents the initial average temperature of the solid-phase transition material at the initial moment.

The phase transition interface conditions for this model are:

6 $$T(x,t) = T_{m} \quad x = x(t), \quad t > 0$$

### Solution of one-dimensional phase change heat transfer based on Lightfoot integral equation

The first part *T*_*1*_(*x,t*) corresponds to the solution of the heat conduction problem without a heat sink, where the boundary conditions and initial conditions remain unchanged. The governing equation for heat transfer is as follows:

7 $$\frac{{\partial^{2} T_{1} (x,t)}}{{\partial x^{2} }} = \frac{1}{\alpha }\frac{{\partial T_{1} (x,t)}}{\partial t} \quad 0 < x < L,t > 0$$

7 $$\alpha = \frac{k}{\rho c}$$

The initial and boundary conditions corresponding to the first part *T*_*1*_(*x,t*) are as follows:

9 $$T_{1} (x,t) = T_{i} \quad 0 < x < L,t = 0$$

10 $$T_{1} (x,t) = T_{B} \quad x = 0,t > 0$$

According to the principles of heat conduction theory, the solution for the first part can be derived as follows^29,30^:

11 $$T_{1} (x,t) = T_{B} + (T_{i} - T_{B} ){\text{erf}}\left( {\frac{x}{{2\sqrt {\alpha t} }}} \right)$$

12 $${\text{erf(x) = }}\frac{2}{\sqrt \pi }\int_{0}^{x} {e^{{ - s^{2} }} } ds$$

The second part* T*_*2*_(*x,t*) corresponds to the solution of a heat conduction problem with a moving heat source, where both the boundary temperature and the initial temperature are zero. The governing heat transfer equation is as follows:

13 $$k\frac{{\partial^{2} T_{2} (x,t)}}{{\partial x^{2} }} + \rho \Delta h\frac{dx(t)}{{dt}}\delta [x - x(t)] = \rho c\frac{{\partial T_{2} (x,t)}}{\partial t} \quad 0 < x < L,t > 0$$

The initial and boundary conditions corresponding to the second part *T*_*2*_(*x,t*) are as follows:

14 $$T_{2} (x,t) = 0 \quad 0 < x < L,t = 0$$

15 $$T_{2} (x,t) = 0 \quad x = 0,t > 0$$

The Green's function method relies on the analytical solution of linear partial differential equations. By constructing a specific Green's function as a fundamental solution, this method facilitates the solution of problems under specified boundary and initial conditions. The computation based on the Green's function method yields:

16 $$T_{2} (x,t) = \frac{\alpha }{k}\int_{\tau = 0}^{t} {d\tau } \int_{x = 0}^{\infty } {G(x,t/x^{\prime},\tau )} g(x^{\prime},\tau )dx^{\prime}$$

In the equation: τ is the dimensionless time; *G*(*x*,*t* / *x′*,*τ*) is represented by the Green's function, and the corresponding expression is:

17 $$G(x,t/x^{\prime},\tau ) = \frac{1}{{2\sqrt {\pi \alpha (t - \tau )} }}\left[ {e^{{ - \frac{{(x - x^{\prime})^{2} }}{4\alpha (t - \tau )}}} - e^{{ - \frac{{(x - x^{\prime})^{2} }}{4\alpha (t - \tau )}}} } \right]$$

The expression for *g*(*x′*,*τ*) is as follows:

18 $$g(x^{\prime},\tau ) = \rho \Delta h\frac{dx(t)}{{dt}}\delta [x^{\prime} - x(t)]$$

The expression for *dx′* after integrating with respect to *T*_*2*_(*x,t*) simplifies to:

19 $$T_{2} (x,t) = \frac{\Delta h}{{2c\sqrt {\pi \alpha } }}\int_{\tau = 0}^{t} {\frac{dx(t)}{{dt}}} \frac{1}{{\sqrt {(t - \tau )} }}\left[ {e^{{ - \frac{{(x - x^{\prime})^{2} }}{4\alpha (t - \tau )}}} - e^{{ - \frac{{(x - x^{\prime})^{2} }}{4\alpha (t - \tau )}}} } \right]d\tau$$

Upon obtaining the expressions for *T*_*1*_(*x*,*t*) and *T*_*2*_(*x*,*t*), their summation yields the expression for the temperature distribution across the entire region:

20 $$T(x,t) = T_{B} + (T_{i} - T_{B} )erf\left( {\frac{x}{{2\sqrt {\alpha t} }}} \right) + \frac{\Delta h}{{2c\sqrt {\pi \alpha } }}\int_{\tau = 0}^{t} {\frac{dx(t)}{{dt}}} \frac{1}{{\sqrt {(t - \tau )} }}\left[ {e^{{ - \frac{{(x - x^{\prime})^{2} }}{4\alpha (t - \tau )}}} - e^{{ - \frac{{(x - x^{\prime})^{2} }}{4\alpha (t - \tau )}}} } \right]d\tau$$

In order to derive the final temperature distribution solution, further integration of the above expression is required. The position of the solid–liquid interface *x*(*t*) can be expressed using the following equation:

21 $$x(t) = 2\lambda \sqrt {\alpha t}$$

Introduce the following two variables:

22 $$y = \frac{x}{x(t)}$$

23 $$\tau = t\left( {\frac{{1 - z^{2} }}{{1 + z^{2} }}} \right)^{2}$$

Replace the limits of integration for *T*_*2*_(*x*,*t*) with variables:

24 $$\left\{ \begin{gathered} \tau = 0 \to z = 1 \hfill \\ \tau = t \to z = 0 \hfill \\ \end{gathered} \right.$$

Further simplification yields:

25 $$T_{2} (x,t) = \frac{2\Delta h\lambda }{{c\sqrt \pi }}(S_{1} - S_{2} )$$

In the equation:

26 $$\left\{ \begin{gathered} S_{1} = \int_{0}^{1} {\frac{dz}{{1 + z}}} e^{{ - \frac{{\lambda^{2} }}{4}\left[ {\frac{y - 1}{z} + (y + 1)z} \right]^{2} }} \hfill \\ S_{2} = \int_{0}^{1} {\frac{dz}{{1 + z}}} e^{{ - \frac{{\lambda^{2} }}{4}\left[ {\frac{y + 1}{z} + (y - 1)z} \right]^{2} }} \hfill \\ \end{gathered} \right.$$

Further manipulation yields the expression for *S*_*1*_ and *S*_*2*_:

27 $$S_{1} = \left\{ \begin{gathered} \frac{\pi }{4}e^{{\lambda^{2} }} {\text{erfc}}(\lambda )[1 + {\text{erf(}}\lambda {\text{y)}}] \quad y < 1 (0 < x < x(t)) \hfill \\ \frac{\pi }{4}e^{{\lambda^{2} }} {\text{erfc}}(\lambda y)[1 + {\text{erf(y)}}] \quad y > 1 (x(t) < x < L) \hfill \\ \end{gathered} \right.$$

28 $$S_{2} = \frac{\pi }{4}e^{{\lambda^{2} }} {\text{erfc}}(\lambda y) {\text{erfc}}(\lambda )$$

In the equation: the complementary error function corresponding to erf(x) is erfc(x), and its expression is:

29 $${\text{erfc(x) = 1 - erf(x) = }}\frac{2}{\sqrt \pi }\int_{x}^{\infty } {e^{{ - \eta^{2} }} } d\eta$$

Further simplification leads to the final form of *T*(*x*,*t*) as follows:

30 $$T(x,t) = \left\{ \begin{gathered} T_{B} { + }(T_{i} - T_{B} ){\text{erf}}\left( {\frac{x}{{2\sqrt {\alpha t} }}} \right) + \frac{\Delta h\lambda \sqrt \pi }{c}e^{{\lambda^{2} }} {\text{erfc(}}\lambda {\text{)erf}}\left( {\frac{x}{{2\sqrt {\alpha t} }}} \right) \quad 0 < x < x(t) \hfill \\ T_{B} { + }(T_{i} - T_{B} ){\text{erf}}\left( {\frac{x}{{2\sqrt {\alpha t} }}} \right) + \frac{\Delta h\lambda \sqrt \pi }{c}e^{{\lambda^{2} }} {\text{erf(}}\lambda {\text{)erfc}}\left( {\frac{x}{{2\sqrt {\alpha t} }}} \right) \quad x(t) < x < L \hfill \\ \end{gathered} \right.$$

Combining the interface condition *x*(*t*) of the solid–liquid interface:

31 $$T_{m} = T_{B} + (T_{i} - T_{B} ){\text{erf(}}\lambda {)} + \frac{\Delta h\lambda \sqrt \pi }{c}e^{{\lambda^{2} }} {\text{erf(}}\lambda {\text{)erfc(}}\lambda {)}$$

Arranging the above equation, the equation for *λ* can be obtained as follows:

32 $$\left[ {\frac{{e^{{ - \lambda^{2} }} }}{{{\text{erf(}}\lambda {)}}} + \frac{{T_{m} - T_{i} }}{{T_{m} - T_{B} }}\frac{{e^{{ - \lambda^{2} }} }}{{{\text{erfc(}}\lambda {)}}}} \right] = \frac{\lambda \Delta h\sqrt \pi }{{c(T_{m} - T_{B} )}}$$

In general, *λ* can only be determined using graphical or iterative methods. Taking into consideration the negligible distinction in the thermal properties between the solid and liquid phases of phase-change materials, namely *k*_*l*_ = *k*_*s*_ and *α*_*l*_ = *α*_*s*_, the computation for λ can be approximated as:

33 $$\lambda \approx \frac{{1}}{{2}}\left[ {2\frac{{c(T_{m} - T_{B} )}}{\Delta h} + \frac{{c^{2} (T_{i} - T_{m} )^{2} }}{{\Delta h^{2} }}} \right]^{\frac{1}{2}} - \frac{{T_{i} - T_{m} }}{{T_{m} - T_{B} }}\frac{{c(T_{m} - T_{B} )}}{\Delta h}$$

### The temperature variation during the phase change heat transfer process.

This article selects the thermal properties of T70# wax to develop a numerical model for one-dimensional phase change heat transfer. See Table 3 for the thermal parameters of T70# wax, and *T*_*i*_ = 20 ℃.

In order to extend the applicability of the theoretical model to other phase transition heat transfer problems or relevant structures, it is imperative to nondimensionalize the variables during the calculation process.

34 $$X = \frac{x}{L}$$

35 $$Fo = \frac{\alpha t}{{L^{2} }}$$

36 $$Ste = \frac{{c(T_{B} - T_{m} )}}{\Delta h}$$

In the equation: *X* represents the ratio of a selected coordinate length to the total thickness of the phase change material; *Fo* denotes the Fourier number, signifying the dimensionless time of the phase change process; *Ste* represents the Stefan number, indicating the ratio of sensible heat to latent heat during the material phase change. Under specific material parameter conditions, the value of *Ste* primarily depends on the magnitude of *T*_*B*_, which in turn is influenced and determined by the external temperature field.

**Figure 6:**
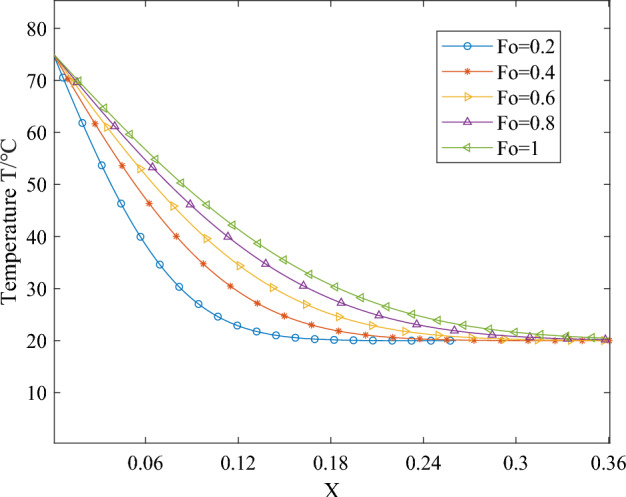
The temperature distribution of PCMs during the melting process with a boundary condition of *Ste* = 0.1.

**Figure 7:**
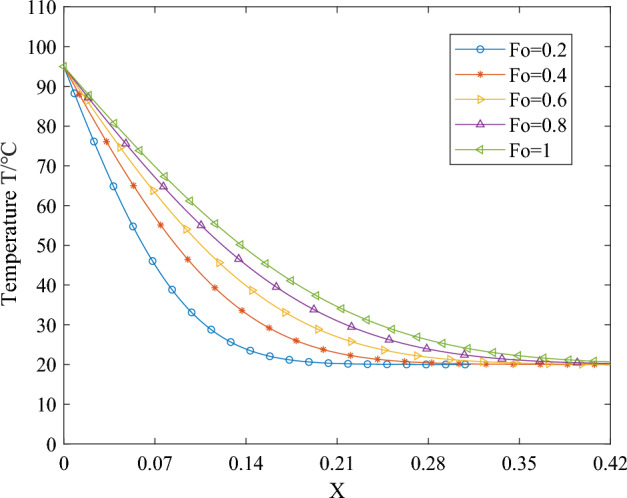
The temperature distribution of PCMs during the melting process with a boundary condition of *Ste* = 0.3.

**Figure 8:**
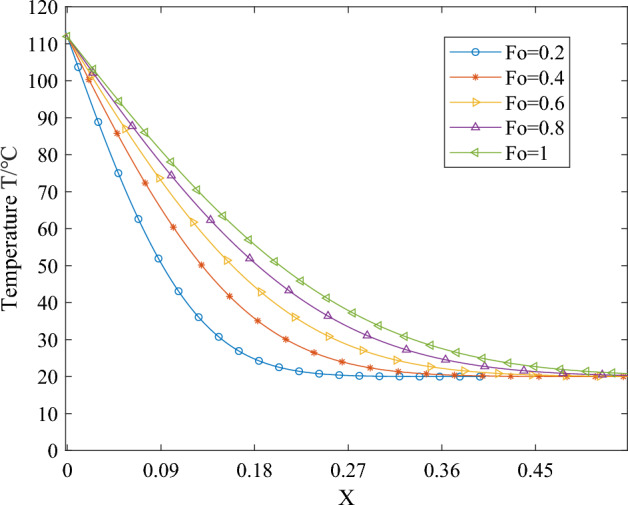
The temperature distribution of PCMs during the melting process with a boundary condition of *Ste* = 0.5

Upon introducing dimensionless parameters and integrating the temperature field expression for one-dimensional phase change heat transfer, the corresponding temperature curves depicting the endothermic melting of the phase change material can be obtained. When *T*_*B*_ is constant, the simplification of *Ste* also yields a constant value. Figures 6, 7 and 8 illustrate the variations in temperature over time and space of the phase change material during the endothermic melting process. Specifically, Fig. 6 presents the simulation results for the boundary condition *Ste* = 0.1 during phase change material melting, Fig. 7 depicts the simulation results for the boundary condition *Ste* = 0.3, and Fig. 8 illustrates the simulation results for the boundary condition *Ste* = 0.5.

From Figs. 6, 7 and 8, it is evident that the increase in *Ste* number signifies a simultaneous rise in sensible heat value while latent heat value remains constant. The escalation in sensible heat value indicates an elevation in the boundary condition temperature *T*_*B*_. Consequently, at the same moment and location, the overall temperature rises correspondingly with the increase in the *Ste* number. Whether in temporal or spatial changes, the temperature trend of the phase-change material at the same moment and location during the heating process remains consistent. In the initial stages of heat transfer, due to the neglect of the influence of liquid-phase material flow in the simulation process, the heat transfer within the material takes the form of pure conduction, with the material's thermal property parameters being constant. Therefore, during the early stages of heating, the temperature variation curve of the phase-change material exhibits an approximately linear change with spatial position.

Comparing the temperature distribution of the phase-change material at the same position under different heating durations with identical boundary conditions reveals distinct patterns: Closest to the heating boundary, the outermost layer of the phase-change material undergoes rapid phase transition from solid to liquid due to the abrupt increase in external temperature. Consequently, the temperature of the liquid-phase material rises swiftly, and the solid–liquid phase interface begins to move away from the boundary. This phenomenon results in more solid-phase material undergoing phase transition and absorbing heat, thereby mitigating the rapid temperature increase during the phase change process. As heating progresses, phase transitions occur successively within the internal phase-changing material, causing the solid–liquid interface to migrate inward.

When researching and utilizing phase change materials, it is imperative to closely monitor the positional alterations of the solid–liquid phase interface. The location of this interface is not only a pivotal conduit for the equilibrium of thermal transmission but also dictates the amount of latent heat that can be absorbed or released by the phase change materials at a specific temperature, thereby influencing their thermal management efficacy in practical applications. By integrating the phase change temperature *T*_*m*_ into the expression for the temperature field:

37 $$T_{m} = T_{B} + (T_{i} - T_{B} ){\text{erf}}\left( {\frac{x}{{2\sqrt {\alpha t} }}} \right) + \frac{\Delta h\lambda \sqrt \pi }{c}e^{{\lambda^{2} }} {\text{erfc}}(\lambda ){\text{erf}}\left( {\frac{x}{{2\sqrt {\alpha t} }}} \right)$$

Further simplification yields:

38 $$\left\{ \begin{gathered} s = \frac{x}{\sqrt t } \hfill \\ T_{m} = T_{B} + (T_{i} - T_{B} ){\text{erf}}\left( {\frac{s}{2\sqrt \alpha }} \right) + \frac{\Delta h\lambda \sqrt \pi }{c}e^{{\lambda^{2} }} {\text{erfc}}(\lambda ){\text{erf}}\left( {\frac{s}{2\sqrt \alpha }} \right) \hfill \\ \end{gathered} \right.$$

In the equation: *s* represents the relationship of the solid–liquid phase interface position with respect to time and space. Under constant boundary conditions, *s* is a constant, while *x* exhibits an exponential function form in relation to *t*. Consequently, when the boundary conditions are fixed, *X* will manifest as an exponential function of Fo.

**Figure 9:**
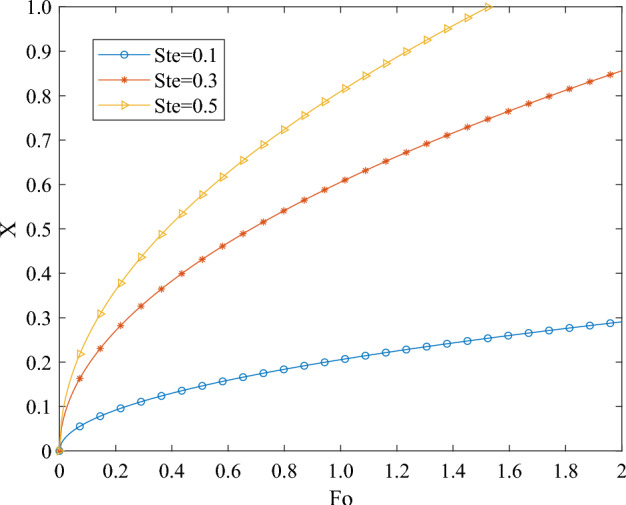
The temporal and spatial variations of the solid–liquid phase interface position.

Figure 9 depicts the variation in the position of the solid–liquid phase interface with respect to time and space during the heat absorption and melting of phase change materials. Due to the entirety of the material being in the solid phase at the initial moment, the starting position of the solid–liquid phase interface on the x-axis moves in the direction of heat transfer in an exponential function form as the phase change process progresses. According to the illustration in Fig. 9, during the initial phase of the heat transfer process, the movement speed of the solid–liquid phase interface is notably swift. This acceleration occurs because at the onset of heat transfer, the outermost layer's temperature rapidly escalates to the phase transition temperature of the phase-change material. At this juncture, the phase-change material initiates its transition into a molten state, absorbing thermal energy primarily to overcome the interparticle attraction within the substance, facilitating the transition from one state to another. Consequently, this prompts a rapid displacement of the interface between the solid and liquid states, creating a pronounced separation from the boundary. As the external heat transfer process progresses, the velocity of the solid–liquid phase interface gradually approaches stability. This is because as the phase change heat absorption of phase change materials continues, the distance between the solid–liquid interface and the boundary surface gradually expands, leading to more solid materials transforming into liquid. This leads to an increase in thermal resistance between the solid–liquid phase interface and the boundary surface, requiring a greater amount of heat absorption to overcome this resistance for further movement of the solid–liquid phase interface. Consequently, the velocity of the solid–liquid phase interface gradually slows down.

Furthermore, a higher *Ste* indicates a greater temperature difference in heat transfer, accelerating the movement of the solid–liquid phase interface. In the context of the melting and heat absorption process of materials, a larger temperature difference implies a higher temperature at the heat transfer boundary. Consequently, the higher the temperature at the heat transfer boundary, the quicker the melting and heat absorption process ensues.

## Experimental analysis

The instrumentation compartment structure was manufactured to ensure it meets the specifications outlined in the drawings. To validate the accuracy of the theoretical model and investigate the temperature variation trends within the instrument compartment, thermal experiments were conducted. A thermal experimental system was established for this structure, encompassing equipment such as heating pads, a PLC control system, temperature sensors, thermocouples, relays, and circuit breakers.

**Figure 10:**
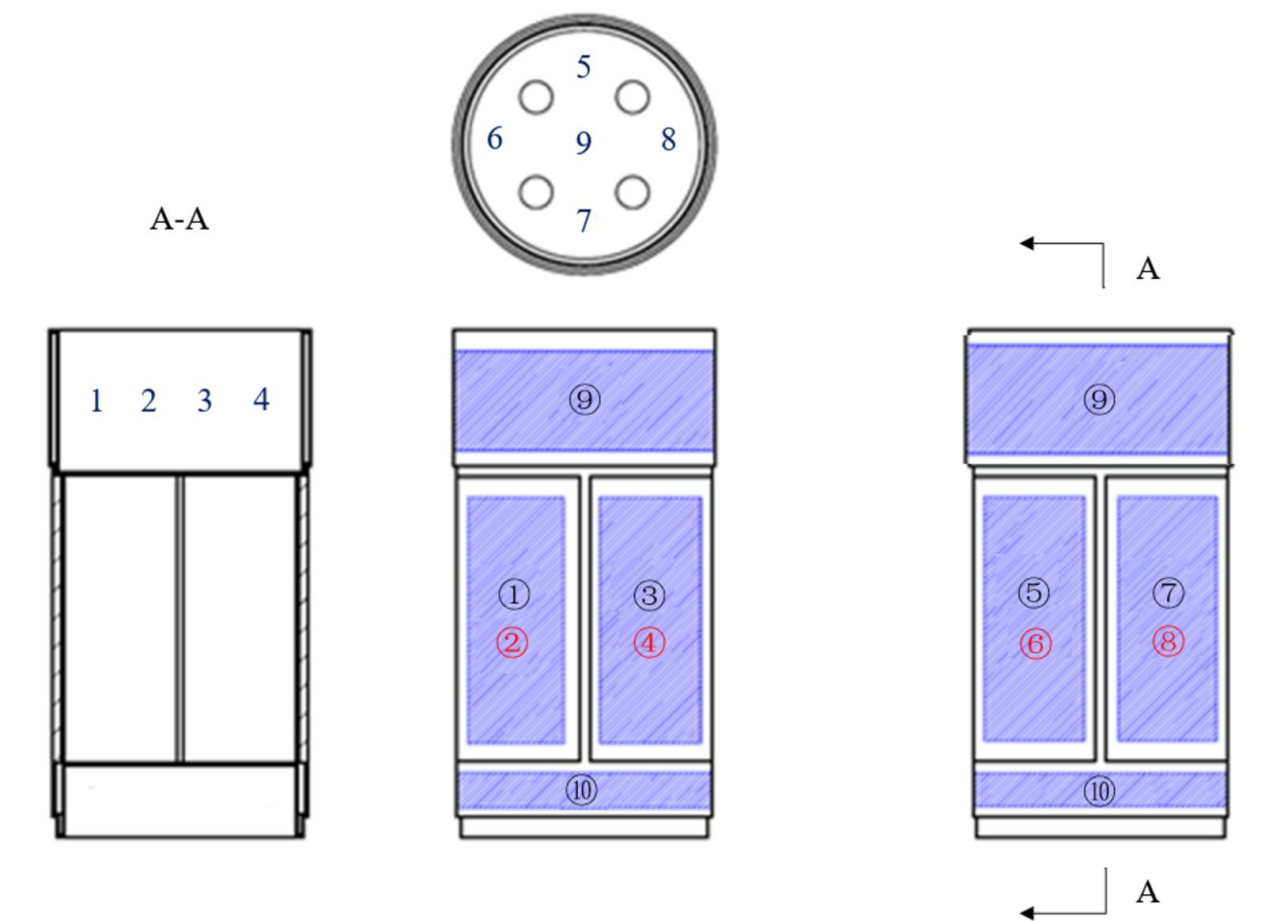
The schematic diagram illustrating the arrangement of temperature measurement points and heating pads.

**Figure 11:**
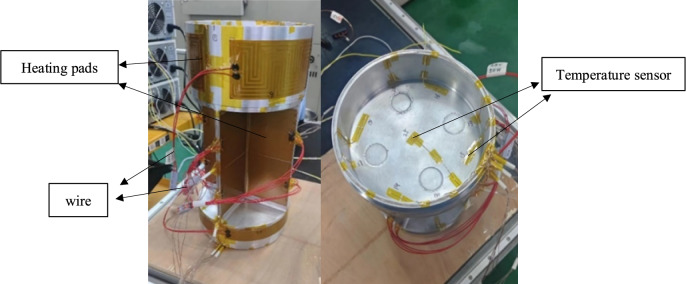
The arrangement of the instrument compartment structure before conducting the experiments.

Ten heating pads are arranged on the outer surface of the instrument compartment, numbered from ① to ⑩. Both sides of the central cross structure are flatly attached with heating pads, while heating film patches ⑨ and ⑩ are respectively arranged on the outer walls of the upper and lower end caps. The dimensions and power of the heating pads are detailed in Table 4. To explore the temperature variations during the heating process of the instrument compartment, nine temperature sensors are strategically positioned for data collection, labeled from 1 to 9. Four temperature measurement points are set on the inner wall of the upper end cap at the middle position, with a quarter-circle interval between adjacent points. Additionally, five temperature measurement points are distributed at the bottom of the upper end cap in a cross-shaped pattern. Figure 10 illustrates the specific locations of temperature measurement points and heating pads arranged on the instrumentation compartment. Figure 11 illustrates The arrangement of experimental equipment within the instrument compartment structure prior to conducting the experiments.

**Table 4:** Heating membrane parameter table.

Heating pad number	Heating pad size (mm)	Heating pad power (W)	Notes
①②③④⑤⑥⑦⑧	210 × 85	100	8 pieces
⑨	620 × 100	150	1 pieces
⑩	600 × 35	150	1 pieces

**Figure 12:**
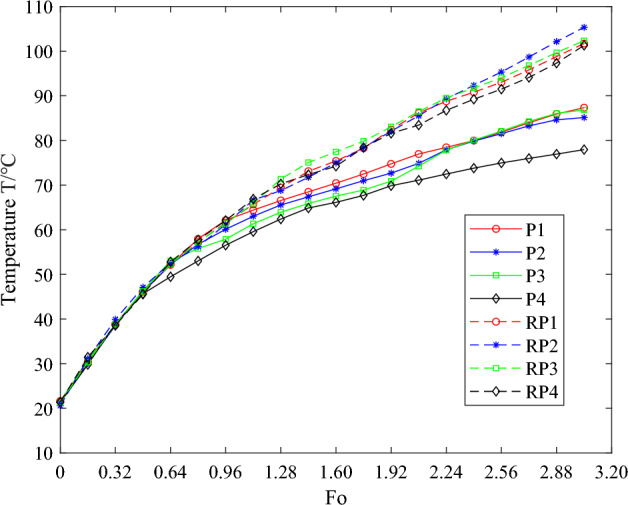
The temperature curve graph for temperature measurement points 1–4 on the inner wall of the upper end cap.

**Figure 13:**
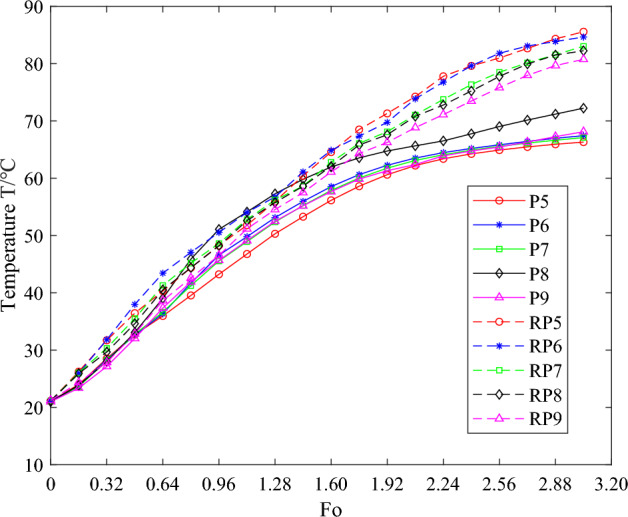
The temperature curve graph for temperature measurement points 5–9 on the inner wall of the upper end cap.

**Figure 14:**
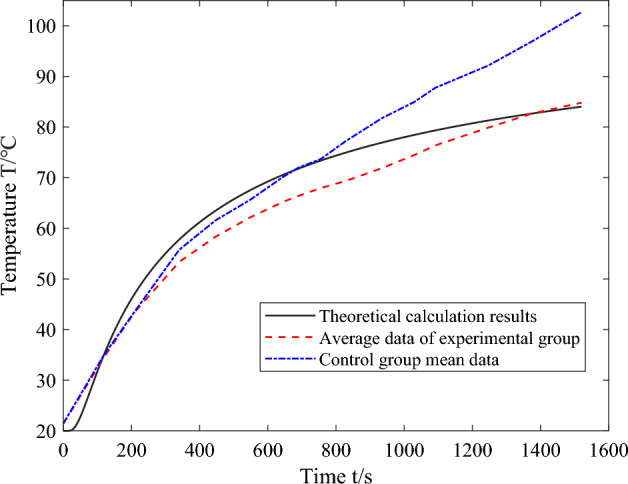
The comparison graph between the mean temperatures of temperature measurement points on the inner edge of the upper end cap and theoretical calculation results.

In the laboratory environment, the equipment was methodically arranged within the structure of the instrument compartment, ensuring the initiation of heating and timing under the premise of experimental safety. Temperature recordings were taken at intervals of 20 s, acquiring experimental data from each measurement point. Concurrently, the recorded data underwent dimensionless processing, culminating in the drafting of temperature curve diagrams. To assess the impact of phase change heat absorption on structural temperature fluctuations, this article employed a control group consisting entirely of instrument compartment structures crafted from 7075 aluminum alloy of matching dimensions, undergoing identical experiments under equivalent conditions. Figures 12 and 13 illustrate the temperature variation curves corresponding to the temperature measurements collected from the upper end cap's inner wall and bottom under two conditions. The dashed lines depict the temperature variations of the instrument compartment structure fabricated entirely from 7075 aluminum alloy. Temperature measurement points 1–4 were used to gather the temperature of the upper end cap's inner wall of the instrument compartment. The temperature measurement point in the instrument compartment structure filled with phase change materials is abbreviated as P, and the corresponding reference temperature measurement point in the control group is abbreviated as RP. In order to enhance data accuracy and mitigate random errors, a mean treatment was applied to the temperature data obtained from these designated measurement points. Figure 14 displays the mean temperature curve at the central position of the inner wall of the upper end cap of the instrument compartment structure filled with phase change materials during the heating process. Herein, the solid blue line represents the outcomes derived from theoretical calculations, serving the purpose of comparison and verification against the results obtained from the established one-dimensional phase change heat transfer theoretical numerical analysis.

The temperature variation curves depicted in Figs. 12 and 13 illustrate that, during the initial stages of heating, the solid T70# wax within the instrument compartment absorbs sensible heat and warms up, without undergoing any phase transformation, leading to an approximately linear temperature increase. Upon the temperature reaching the phase change threshold due to heating, the T70# wax commences its gradual transition from solid to liquid state, accompanied by the endothermic process of phase change, which decelerates the rate of temperature rise, eventually stabilizing slowly and steadily. A comparative analysis between the instrument compartment structure made entirely of 7075 aluminum alloy and that incorporating phase change materials reveals that, in the early stages of heating, the temperature trends of both structures are largely similar, with no significant divergence. However, in the later stages of heating, the temperature at the same moment and location within the structure containing phase change materials is noticeably lower than that of the pure aluminum alloy structure, with a more gradual rate of temperature increase. This indicates that the instrument compartment structure filled with phase change materials effectively delays the rise in its structural temperature by absorbing heat, thereby possessing a commendable thermal control function. The bottom of the upper end cap of the instrument compartment structure, filled with phase change materials, is situated at a considerable distance from the heat source. Consequently, the temperatures measured at this point are slightly lower than those along the inner wall of the upper end cap. Nonetheless, both exhibit similar overall trends: an initial ascent in temperature, followed by a gradual increase, ultimately stabilizing.

The mean data collected from the aluminum alloy instrument compartment structure in Fig. 14 are significantly higher than the other two sets of data, indicating that the instrument compartment structure filled with phase change materials can provide a certain degree of thermal protection during the heat transfer process. This is because the phase change materials filled inside can absorb heat during the phase change process, effectively slowing down the rate of temperature increase of the structure itself. Figure 14 illustrates that the experimental mean values are, on the whole, slightly lower when compared with theoretical calculations. The underlying reason for this discrepancy can be attributed to the theoretical calculations primarily focusing on the temperature of the phase change material's boundary surface, whereas the experimental measurements pertain to the inner wall temperature of the casing, not directly measuring the actual temperature within the phase change material. There exists a certain thickness of an aluminum alloy casing between the experimental measurement points and the theoretical calculation points, which may have created a temperature gradient along the thickness of the casing. Moreover, external factors such as ambient temperature during the measurement process could also lead to a lower measured temperature. When viewed overall, the mean temperature curve is in good agreement with the theoretical calculation, being relatively close in numerical terms. This demonstrates that the one-dimensional phase change heat transfer model can describe the temperature variations within the instrument compartment structure during the phase change process with a relative degree of accuracy, thereby validating the reliability of the theoretical model in predicting the phase transition of materials.

## Conclusions

This article focuses on the structural model of an instrumentation compartment filled with phase change materials. A one-dimensional model for phase change heat transfer was developed, followed by an analysis of the one-dimensional phase change heat transfer mechanism and thermal experiments. The following conclusions were drawn:

1. The proposed instrumentation compartment design scheme based on phase change materials is characterized by its simplicity in design, lightweight construction, absence of additional volume occupancy requirements, and reusability.
2. Based on the characteristics of the instrument compartment structural model, a one-dimensional phase-change heat conduction theoretical model is constructed in conjunction with the Lightfoot integral equation method. The model is numerically solved using the Green's function method. The theoretical calculations obtained align well with the results of thermal experiments, verifying that this one-dimensional phase-change heat transfer model possesses commendable accuracy and reliability.
3. In comparison to the instrument compartment structure comprised solely of 7075 aluminum alloy, the incorporation of phase change materials within the structure facilitates the absorption of heat during the phase change process. This effectively reduces the intrinsic temperature of the structure, thereby accomplishing a degree of thermal management.

## Discussion

This paper selects T70# wax as the material, mainly considering two requirements when designing the instrument compartment structure: the temperature of the phase change heat load when charging the phase change medium is about 70 °C, and the phase change storage heat is about 170 kJ. Taking these two factors into account, we looked for suitable phase change materials among corresponding organic and inorganic phase change materials. Additionally, considering the need for scientific research trials to have controllable research costs, T70# wax was ultimately chosen. If the corresponding working force/thermal environment is different, and a higher phase change temperature and stronger carrying capacity are required, high-temperature phase change materials such as metal carbonates, fluorides, nitrates (with phase change temperatures above 500 °C) and high-strength alloys such as titanium alloys and other high-strength alloys should be selected.

For different mission objects or working environments, when selecting phase change materials, it is necessary to consider the relevant design requirements: such as the temperature range for thermal protection and the amount of heat that needs to be absorbed by the protected area. Taking batteries as an example, to prevent high temperatures from affecting the performance of the batteries, the phase change temperature of the phase change material is generally chosen to be close to the highest temperature of the batteries. This allows it to absorb heat and reduce the temperature peak of the batteries during phase change, achieving the effect of protecting the batteries. Generally, the highest temperature of batteries is between 60 and 100 °C, so phase change materials with corresponding phase change temperatures should be selected. Similarly, satellite thermal protection temperatures can reach above 200 °C, so high-temperature phase change materials should be chosen according to different needs.

## Data Availability

The datasets generated during and/or analysed during the current study are available from the corresponding author on reasonable request.

## List of symbols

*E*: Elastic modulus (GPa)
*μ*: Poisson's ratio
*ρ*: Density (kg/m^3^)
*c*: Specific heat capacity (J kg^−^^1^ ℃^−^^1^)
*k*: Thermal conductivity coefficient (W m^-1^ ℃^-1^)
*α*: Coefficient of thermal expansion (℃^-1^)
*σ*_*s*_: Yield limit (MPa)
*T*_*m*_: Phase transition temperature (℃)
*T*_*B*_: Boundary temperature (℃)
*T*_*i*_: Initial average temperature of the solid-phase transition material at the initial moment (℃)
Fo: Fourier number, signifying the dimensionless time of the phase change proces
Ste: Stefan number
